# Bioefficacy of *Rhynocoris longifrons* (Stål) (Heteroptera: Reduviidae) against multiple cotton pests under screen house and field conditions

**DOI:** 10.1038/s41598-020-63768-0

**Published:** 2020-04-20

**Authors:** Kitherian Sahayaraj, Subramanian Kalidas, Loko Yêyinou Laura Estelle

**Affiliations:** 1 Crop Protection Research Centre, Dept. of Zoology, St. Xavier’s College, Palayamkottai-627 002, Tamil Nadu, India; 2 Ecole Nationale Supérieure des Biosciences et Biotechnologies Appliquées (ENSBBA), Université Nationale des Sciences, Technologies, Ingénierie et Mathématiques (UNSTIM), BP 14, Dassa, Benin

**Keywords:** Ecology, Plant sciences, Zoology, Ecology, Environmental sciences, Natural hazards, Health care

## Abstract

*Rhynocoris longifrons* (Hemiptera: Reduviidae) is a generalist predator of many cotton insect pests. The hiding behaviour of this predator, which is one of the key factors of predation success, was investigated under screen house conditions. Moreover, we evaluated its biocontrol potential against *Aphis gossypii* (Hemiptera: Aphididae), *Dysdercus cingulatus* (Hemiptera: Pyrrhocoridae), *Phenacoccus solenopsis* (Hemiptera: Pseudococcidae), and *Helicoverpa armigera* Hübner (Lepidoptera: Noctuidae) under screen house and field conditions. Results showed that *R. longifrons* life stages preferred to hide under small pebbles in the screen house tests. All the *R. longifrons* life stages showed a biocontrol potential against the four insect pests under screen house conditions. However, their biocontrol potential had not varied in relation to day and night hours. Augmentative releases of *R. longifrons* were carried out for two seasons such as South-west monsoon, 2011 and post-monsoon, 2012. The augmentative release of *R. longifrons* reduced significantly insect pests on cotton. In fact, the release of this predator in cotton fields was capable to reduce the population of *H. armigera* (50%), *P. solenopsis* (28%), *D. cingulatus* (18.8%), and *A. gossypii* (11.8%) during the rain fed condition (south-west monsoon season). During irrigated condition (post-monsoon season), populations of *D. cingulatus* were reduced by 26%, than *P. solenopsis* (20.6%), and *A. gossypii* (16.8%). Except ants, no negative impact was reported on other natural enemies present in the cotton field. Significantly higher crop yield and cost benefit ratio was observed in the predator release plots indicating that *R. longifrons* can be used in an integrated pest management program for multiple cotton pests.

## Introduction

Cotton (*Gossypium hirsutum* L., Malvaceae), is an important cash crop throughout the world. However, its production is severely hampered by several abiotic and biotic factors, such as insect attacks that lead to significant yield reduction. *Aphis gossypii* Glover (Hemiptera: Aphididae)^1^, *Dysdercus cingulatus* (Fab.) (Hemiptera: Pyrrhocoridae)^2^, *Phenacoccus solenopsis* Tinsley (Hemiptera: Pseudococcidae)^3^, and *Helicoverpa armigera* Hübner (Lepidoptera: Noctuidae)^4^ are considered as economically important pests of cotton. Conventional synthetic chemical insecticides are typically extensively used causing significant side effects including pesticide resistance^5^ as well as having many ecological and biological impacts^6^.

Members of Reduviidae are abundant predators of many economically important insect pests^7–9^. Reduviids are common in cotton agro-ecosystems^10,11^. However, they often fail to colonize fields to provide effective control of the pests. In such a situation, augmentative biological control can be an important approach to protect the cotton. Augmentative biological control is practiced worldwide with more than 150 species of natural enemies now commercially available^12^. However, generalist predators, particularly predatory bugs, have been largely ignored for augmentative biological control of cotton pests^13,14^.

A number of researches investigated the impact of augmentative release of various reduviids against a wide variety of insect pest’s world-wide^15–20^. However, in cotton growing regions of India, biocontrol potential of reduviids have not fully explored. Native reduviid predator species have shown good predation against many insect pests^8,21^. One of the most important genera of Reduviidae, as well as widely present in many agro-ecosystems is *Rhynocoris* Hahn (Hemiptera: Reduviidae)^9^. Specifically, *Rhynocoris longifrons* (Stål) is a general predator of many insect pests in cotton fields such as: Hemiptera [*A. gossypii*, *D. cingulatus*, *P. solenopsis*^22^, *Clavigralla gibbosa* Spinola^23^ and, *Nezara viridula* Linnaeus^24^]; and Lepidoptera [*H. armigera* and, *Spodoptera litura* Fab.^24^].

Many reduviid predators possess either morphological adaptive characters^25^ or behavioural adaptive features^26^ including hiding^27^ to successfully capture and feed the preys’. Shelter provisioning with pieces of clay pots and stones in cotton field enhanced reduviids population and increased cotton production^21^. To date, specific information on the predatory behaviour and biological control potential of *R. longifrons* against any pests under field conditions has not been widely reported. The predator can exhibit a variety of necessary adaptations for predation as well as survival^28^. One such important adaptation is a hiding behavior, either to escape from natural enemies and/or to find shelter. This hiding behavior, referred to as anti-predator behaviour^29^ has not been studied with reduviid predators. Moreover, Ambrose^30^ reported that reduviid predators of the subfamily Ectrichodiinae are diurnal, whereas Peiratinae and Emesinae are nocturnal. To date, however, no systematic study has been made of the feeding potential of these predatory *R. longifrons* in relation to day and night hours. Therefore, we conducted a series of studies to assess their possible integration in an augmentative biological control program for cotton pests in Tamil Nadu, India. The specific objectives of this study were to: 1) understand *R. longifrons* hiding behaviour under open field conditions, 2) evaluate their biocontrol potential in relation to day and night hours, 3) test their efficacy in an augmentative release program under field conditions for two seasons, and 4) estimate the cost benefit ratio and percent avoidable loss.

## Results

### Hiding behaviour of the predator under screen house

**Figure 1:**
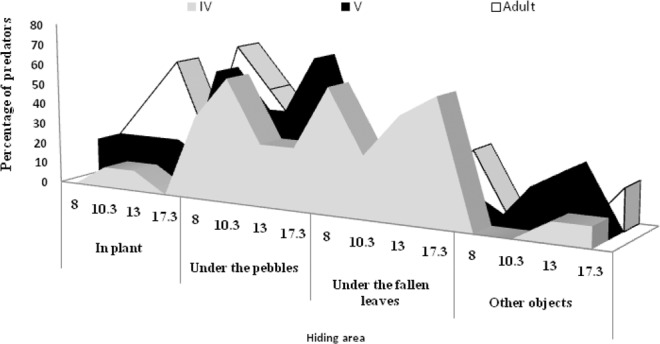
Hiding area of R. longifrons fourth and fifth stadium nymphs and adult (male and female) (%) released from morning 6 a.m., 8.30 a.m., 11.00 a.m., and to 3.30 p.m. and observation made in 2-hours intervals under screen house conditions.

The hiding behaviour of *R. longifrons* revealed that the percentage of predator adults hiding under pebbles was higher at 6 a.m. (*F* = 42.53; df = 3; *P* < 0.000) and 10.30 a.m. (*F* = 8.60; df = 3; *P* < 0.05) than other hiding places (Fig. 1). As day light increased, the predator moved under the plants (*F* = 41.60; df = 3; *P* < 0.005) and then moved either under pebbles (*F* = 8.63; df = 3; *P* < 0.05) or under the fallen leaves (*F* = 8.62; df = 3; *P* < 0.05) for hiding. Fifth-instar reduviids preferred to hide under pebbles (*F* = 42.63; df = 3; *P* < 0.0005), whereas fourth-instar (*F* = 8.60; df = 3; *P* < 0.05) predator first hid under fallen leaves and latter moved into pebbles, again left the place and returned to the fallen leaves (*F* = 8.61; df = 3; *P˂*0.05) to hide (Table 1).

**Table 1:** Hiding location selection (%) of *R. longifrons* life stages released under screen house conditions (n = 10).

Predator life stages	Observed percentage of predator life stages in different hiding areas (Mean ± SE)			
	In plants	Under the pebbles	Under the fallen leaves	Other objects
Fourth instar	5.0 ± 1.9c	40.0 ± 3.0b	50.0 ± 7.0a	5.0 ± 1.9c
Fifth instar	17.5 ± 2.5b	52.5 ± 4.5a	15.0 ± 2.9c	12.5 ± 2.5d
Adults (male + female)	35.0 ± 9.5b	45.0 ± 3.5a	15.0 ± 3.0c	5.0 ± 2.0d

Percentage followed by different letters within the same line is significantly different at 0.05 level as determined by the SNK test.

### Biocontrol potential of *R. longifrons* in relation to day and night hours

*Rhynocoris longifrons* life stages significantly consumed more *P. solenopsis* (F = 32.563, df = 1, p ≤ 0.000) and *A. gossypii* adults (F = 6.696, df = 1, p ≤ 0.05), *D. cingulatus* nymphs (F = 49.439, df = 1, p ≤ 0.000), and *H. armigera* larvae (F = 40.119, df = 1, p ≤ 0.000) compared to the control category. No significant differences in the predation rate of *R. longifrons* life stages was recorded on *A. gossypii* (F = 1.022, df = 23, p ≥ 0.05), *D. cingulatus* (F = 1.410, df = 23, p ≥ 0.05), *P. solenopsis* (F = 0.794, df = 23, p ≥ 0.05), and *H. armigera* (F = 1.092, df = 23, p ≥ 0.05) during dawn hours. Similarly, there was also not much significant difference in the activity of *R. longifrons* life stages on *D. cingulatus* (F = 0.667, df = 23, p ≥ 0.05)*, P. solenopsis* (F = 0.426, df = 23, p ≥ 0.05)*, H. armigera* (F = 2.037, df = 23, p ≥ 0.05), and *A. gossypii* (F = 1.098, df = 23, p ≥ 0.05) during the dusk hours. A similar trend was observed when compared predation rate between dawn and dusk hours of *R. longifrons* life stages on the four preys species (Table 2). When we considered total predation rate (dawn and dusk hours), no significant difference of predation rate was observed between *R. longifrons* life stage when feeding on *D. cingulatus* (F = 1.422, df = 47, p ≥ 0.05)*, P. solenopsis* (F = 1.081, df = 47, p ≥ 0.05), and *H. armigera* (F = 0.839, df = 47, p ≥ 0.05). However, *A. gossypii* was significantly consumed more preys by predator third-instar (F = 3.017, df = 47, p ≤ 0.05).

**Table 2:** Biocontrol potential of *R. longifrons* third, fourth and fifth stadium and adult (male and female) against *A. gossypii*, *D. cingulatus*, *H. armigera*and *P.solenopsis*in relation to dawn and dusk hours (Mean ± SE).

Pest species	Stage of predator	Predator releasing time (mean ± SE)		Total mean	Anova between dawn and dusk releases
		Dawn hours	Dusk hours		
***A. gossypii***	Third instar	0.83 ± 0.31a	0.50 ± 0.34a	0.67 ± 0.22b	
	Fourth instar	0.25 ± 0.17a	0.50 ± 0.31a	0.38 ± 0.18ab	Df = 1
	Fifth instar	0.28 ± 0.18a	0.00 ± 0.00a	0.14 ± 0.09a	F = 0.316
	Adult	0.05 ± 0.05a	0.11 ± 0.11a	0.08 ± 0.06a	P = 0.575
***D. cingulatus***	Third instar	0.25 ± 0.17a	0.33 ± 0.10a	0.29 ± 0.09a	
	Fourth instar	0.17 ± 0.10a	0.25 ± 0.11a	0.21 ± 0.07a	Df = 1
	Fifth instar	0.33 ± 0.08a	0.44 ± 0.14a	0.39 ± 0.08a	F = 0.216
	Adult	0.49 ± 0.9a	0.72 ± 0.10a	0.61 ± 0.11a	P = 0.145
***H. armigera***	Third instar	0.41 ± 0.15a	0.67 ± 0.16a	0.54 ± 0.11a	
	Fourth instar	0.50 ± 0.18a	1.08 ± 0.35a	0.79 ± 0.21a	Df = 1
	Fifth instar	0.55 ± 0.14a	0.38 ± 0.13a	0.47 ± 0.09a	F = 0.013
	Adult	0.88 ± 0.13a	0.49 ± 0.20a	0.68 ± 0.13a	P = 0.909
***P. solenopsis***	Third instar	0.91 ± 0.15a	1.00 ± 0.22a	0.96 ± 0.13a	
	Fourth instar	1.00 ± 0.28a	0.75 ± 0.17a	0.87 ± 0.13a	Df = 1
	Fifth instar	0.65 ± 0.26a	0.61 ± 0.23a	0.63 ± 0.17a	F = 0.028
	Adult	0.82 ± 0.25a	0.82 ± 0.32a	0.82 ± 0.19a	P = 0.867

Percentages followed by different letters within the same line are significantly different at 0.05 level as determined by the Student-Newman-keuls test.

### Bioefficacy of the predators under cotton field conditions

In the cotton fields, when the total number of insect pests was considered, the most predominant insect pests were *A. gossypii* (81%), *P. solenopsis* (10%), *D. cingulatus* (6%), and *H. armigera* (3%), during rain fed condition. However, during irrigated condition, the predominant insect pest was *P. solenopsis* (60.2%), *A. gossypii* (32.8%) and *D. cingulatus* (8.2%). Therefore, we mainly concentrated on these pests throughout our observations. A significant reduction in *A. gossypii* population was observed in *R. Longifrons-*released plots compared to control after the first (F = 58.571, df = 299, p ≤ 0.000) and second (F = 22.415, df = 299, p ≤ 0.000) predator releases during south-west monsoon (Table 3). Yet, during the post-monsoon season a significant reduction in *A. gossypii* population was observed in *R. Longifrons-*released plots after the first (F = 22.761, df = 299, p ≤ 0.0005) and third (F = 5.596, df = 299, p ≤ 0.05) predator releases. During the rain fed condition, significant reduction of *D. cingulatus* (F = 6.951, df = 249, p ≤ 0.05) and *P. solenopsis* (F = 32.147, df = 299, p ≤ 0.0005) populations were recorded respectively during the first and third releases of *R. longifrons* life stages. A significantly lower incidence of *H. armigera* (F = 19.930, df = 299, p ≤ 0.000) was reported only during the first release of *R. longifrons* life stages.

**Table 3:** Effect of augmentative releases of *Rhynocoris longifrons* life stages on the four predominant pest in the cotton fields during South-west monsoon (S-WM, July to September 2011) and Post-monsoon season (P-M, December to February 2011–12) at Virudhunagar and Tuticorin districts respectively.

Number of releases	Seasons	Field treatment	Predominant pests (mean number/plant ± SE)			
			*A. gossypii*	*D. cingulatus*	*P. solenopsis*	*H. armigera*
First release	S-WM	Control	112.81 ± 5,74a	1.13 ± 0.18a	6.63 ± 0.82a	0,19 ± 0.04a
		*R. longifrons* release	73.49 ± 6.37b	2.00 ± 0.39a	1.56 ± 0.33b	0.00 ± 0.00b
	P-M	Control	74.79 ± 3.81a	-	12.71 ± 2.09a	-
		*R. longifrons* release	51.54 ± 3.14b	-	7.25 ± 0.76a	-
Second release	S-WM	Control	98.35 ± 5.62a	0.15 ± 0.04a	0.96 ± 0.21a	0.01 ± 0.00a
		*R. longifrons* release	72.52 ± 3.16b	0.11 ± 0.03a	0.45 ± 0.11a	0.01 ± 0.00a
	P-M	Control	46.19 ± 2.55a	-	8.86 ± 1.11a	-
		*R. longifrons* release	46.64 ± 2.15a	-	7.85 ± 0.82a	-
Third release	S-WM	Control	78.77 ± 3.15a	0.17 ± 0.04a	7.65 ± 1.47a	-
		*R. longifrons* release	68.13 ± 2.92a	0.05 ± 0.02b	7.93 ± 1.51a	-
	P-M	Control	36.85 ± 1.93a	0.17 ± 0.07a	9.09 ± 0.91a	-
		*R. longifrons* release	30.25 ± 1.81 b	0.08 ± 0.05a	9.38 ± 0.84a	-
Total mean population after release	S-WM	Control	96.64 ± 2.94a	0.72 ± 0.13a	5.08 ± 0.58a	0.06 ± 0.01a
		*R. longifrons* release	71.38 ± 2.55b	0.48 ± 0.06a	3.31 ± 0.54b	0.00 ± 0.00b
	P-M	Control	52.61 ± 1.81a	0.06 ± 0.02a	10.22 ± 0.85a	-
		*R. longifrons* release	42.81 ± 1.48b	0.03 ± 0.01a	8.16 ± 0.47a	-

Means followed by different letters in a column for each release and season or the total mean population are significantly different (SNK test, P < 0.05).

During the irrigated condition, a low *D. cingulatus* population appeared in cotton fields after the first release of *R. longifrons* life stages (Table 3). However, no significant reduction of *D. cingulatus* population (F = 0.000, df = 299, p ≥ 0.05) was observed in *R. Longifrons-*treated plots compared with the control. Similarly, during post-monsoon season no significant reduction of *P. solenopsis* population was observed after the first (F = 0.020, df = 299, p ≥ 0.05), second (F = 0.000, df = 299, p ≥ 0.05) and, third (F = 1.198, df = 299, p ≥ 0.05) releases of the predator life stages. When we take in account the total mean population after all three-predator releases, there was a significant reduction of *A. gossypii* (F = 41.908, df = 899, p ≤ 0.000), *P. solenopsis* (F = 4.949, df = 899, p ≤ 0.05), and *H. armigera* (F = 12.734, df = 899, p ≤ 0.000) during south-west monsoon. While, only *A. gossypii* population was significantly reduced after release of *R. longifrons* life stage during post-monsoon season (F = 12.167, df = 899, p ≤ 0.05).

**Figure 2:**
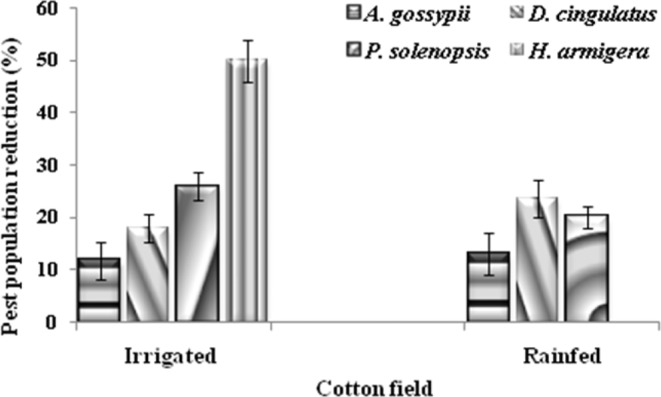
Augmentative releases of *R. longifrons* on the natural enemies population (number/plant) under rainfed condition. Plots without reduviid predator (**A**) and Plots without reduviid predator (**B**).

In general, the release of *R. longifrons* in cotton fields was capable to reduce the populations of *H. armigera* (50%), *P. solenopsis* (28%), *D. cingulatus* (18.8%), *A. gossypii* (11.8%) during south-west monsoon season (Fig. 2). However, during post-monsoon season, populations of *D. cingulatus* were reduced by 26%, followed *P. solenopsis* (20.6%) and *A. gossypii* (16.8%).

### Effect on natural enemies’ populations

Other natural enemies like ants, coccinellids, wasps, other reduviids, and spiders were observed in the cotton fields. During the rainy season, the populations of reduviids (F = 13.223, df = 299, p ≤ 0.000), spiders (F = 20.926, df = 299, p ≤ 0.000), and coccinellids (F = 4.015, df = 299, p ≤ 0.05) had significantly increased in *R. Longifrons-*treated plots from the first and second releases respectively compared to control (Table 4). However, a significant reduction of ants (F = 4.056, df = 299, p ≤ 0.05) and wasps (F = 5.279, df = 299, p ≤ 0.05) populations was observed after the second release of *R. longifrons* life stages. Similar trend was also observed during post-monsoon season where significant reduction of ants (F = 4.983, df = 299, p ≤ 0.05) and wasps (F = 4.388, df = 299, p ≤ 0.05) populations was observed after the third release of *R. longifrons* life stages.

**Table 4:** Effect of augmentative releases of *Rhynocoris longifrons* life stages on natural enemies’ population in the cotton fieldsduring South-west monsoon (S-WM, July to September 2011) and Post-monsoon season (P-M, December to February 2011–12) at Virudhunagar and Tuticorin districts respectively.

Number of releases	Seasons	Field treatment	Mean number ± SE of natural enemies/plant				
			Coccinellids	Ants	Reduviids	Spiders	Wasps
First release	S-WM	Control	0.23 ± 0.05a	0.67 ± 0.10a	0.00 ± 0.00b	0.09 ± 0.04b	0.07 ± 0.02a
		*R. longifrons* release	0.24 ± 0.05a	0.25 ± 0.06b	0.12 ± 0.03a	0.45 ± 0.08a	0.09 ± 0.02a
	P-M	Control	0.45 ± 0.06a	0.86 ± 0.13a	0.00 ± 0.00b	0.11 ± 0.28a	0.09 ± 0.02a
		*R. longifrons* release	0.31 ± 0.02a	0.60 ± 0.11a	0.27 ± 0.08a	0.12 ± 0.02a	0.10 ± 0.03a
Second release	S-WM	Control	0.27 ± 0.04b	0.40 ± 0.06a	0.01 ± 0.01a	0.21 ± 0.03a	0.33 ± 0.04a
		*R. longifrons* release	0.38 ± 0.04a	0.27 ± 0.04b	0.04 ± 0.02a	0.24 ± 0.01a	0.21 ± 0.03b
	P-M	Control	0.31 ± 0.01a	0.64 ± 0.13a	0.00 ± 0.00a	0.12 ± 0.03a	0.04 ± 0.01a
		*R. longifrons* release	0.19 ± 0.04a	0.53 ± 0.10a	0.01 ± 0.00a	0.08 ± 0.02a	0.03 ± 0.15a
Third release	S-WM	Control	0.44 ± 0.05a	0.70 ± 0.06a	0.01 ± 0.01a	0.40 ± 0.04a	0.26 ± 0.04a
		*R. longifrons* release	0.53 ± 0.01a	0.89 ± 0.07a	0.03 ± 0.01a	0.41 ± 0.04a	0.27 ± 0.03a
	P-M	Control	0.32 ± 0.04a	0.68 ± 0.09a	0.00 ± 0.00a	0.21 ± 0.04a	0.14 ± 0.03a
		*R. longifrons* release	0.33 ± 0.05a	0.46 ± 0.07b	0.01 ± 0.00a	0.13 ± 0.02a	0.07 ± 0.02b
Total mean population after release	S-WM	Control	0.32 ± 0.35b	0.59 ± 0.04a	0.01 ± 0.04b	0.23 ± 0.02b	0.18 ± 0.01a
		*R. longifrons* release	0.38 ± 0.30a	0.47 ± 0.38b	0.06 ± 0.01a	0.38 ± 0.03a	0.23 ± 0.02a
	P-M	Control	0.36 ± 0.03a	0.73 ± 0.07a	0.00 ± 0.00b	0.15 ± 0.02a	0.09 ± 0.01a
		*R. longifrons* release	0.28 ± 0.03a	0.53 ± 0.05b	0.09 ± 0.03a	0.11 ± 0.01a	0.07 ± 0.01a

Means followed by different letters in a column for each release and season or the total mean population are significantly different (SNK test, P < 0.05).

When we considered the total mean population after the three releases, there were significant increasing in the population of coccinellids (F = 3.906, df = 899, p ≤ 0.05), reduviids (F = 14.206, df = 899, p ≤ 0.000) and, spiders (F = 8.171, df = 899, p ≤ 0.05) compared with the control plots during south-west monsoon. It is the same trends for the reduviids populations (F = 14.764, df = 899, p ≤ 0.000) during post-monsoon season (Table 4). However, ants populations were significantly reduced as well during south-west monsoon (F = 5.403, df = 899, p ≤ 0.05) than post-monsoon seasons (F = 5.700, df = 899, p ≤ 0.05) in *R. longifrons* treated plots.

### Cotton production, Cost Benefit Ratio (CBR) and Percent avoidable loss

Cotton production was higher in the predator-release plots (837.0 and 753.4 kg /Hectare^−1^ for south-west monsoon and post-monsoon seasons respectively). Similarly, the cost benefit ratio was higher in the predator-release treatment (1:1.28) than in the control treatment (1:1.17) during the south-west monsoon season, as well as post-monsoon (Table 5). Cost of cultivation was less in the control [= 4872.0 (73,08 US Dollar) and 26652.0 (399,78 US Dollar) for South-west monsoon and post-monsoon seasons respectively)] compared with the predator-released field [= 29096.0 (436,44 US Dollar) and 28134.0 (422,01 US Dollar), for South-west monsoon and post-monsoon seasons respectively)]. A higher PAL was recorded in the South-west monsoon season (14.5%) than in the post-monsoon season (4.9%) (Table 5).

**Table 5:** Augmentative release of *R. longifrons* on cost benefit ratio analysis under irrigated and rainfed cotton cultivation.

Expenses (in US dollar)	Field conditions			
	Irrigated condition		Rain fed condition	
	Treatment	Control	Treatment	Control
Plough	54.07	54.07	36.06	36.06
Sowing	29.68	29.68	25.23	25.23
Manure	84.28	84.28	45.05	45.05
Harvesting	55.46	55.46	54.07	54.07
Transportation	—	—	58.05	58.05
Weeding	86.51	86.51	90.11	90.11
Cost of cultivation in *R. longifrons*	21.07	—	21.07	—
Total expenses	331.07	310.00	329.64	308.56
Total Income	424.61	362.97	410.57	388.95
Cotton production (Kg/h^−1^)	837.0	715.5	753.35	716.30
Cost Benefit Ratio (CBR)	1.28	1.17	1.24	1.26
The percent avoidable loss (%)	14.5		4.9	

## Discussion

Reduviids are distributed in cotton agro-ecosystems worldwide and considered as important biocontrol agents^10^. Hence, use of reduviid predators to manage cotton pests can be one of the effective components of IPM programme, thus basic information is needed about hiding behavior, predatory potential, and other factors. The present study clearly shows that *R. longifrons* hide under different objects and, the preference for an object is function of the time of the day. The reduviid predator *Rhynocoris fuscipes* (Fabricius) also showed similar hiding behavior^31^. This hiding behavior is an advantageous in the framework of an IPM because it allows this predator to better surprise the prey it captures. Moreover, it allows a better escaping from their natural enemies such spiders and wasps^31–33^. Therefore, we can conclude that *R. longifrons* nymphs and adults can survive and adapt in the cotton agro-ecosystem after its release.

Our results showed that, feeding potential of *R. longifrons* life stages on *P. solenopsis* and *A. gossypii* adults, *D. cingulatus* nymphs, and *H. armigera* larvae had not varied in relation to day and night hours. This result was surprising because some authors suggested that reduviid predators are either nocturnal or diurnal^34^. For example, Lira *et al*.^35^ found immature Harpactorini assassin bug feeding on a scorpion during the night time only. In contrast, *Sycanus* sp. and *Scipinia* sp. life stages were more active in the morning (6.30 to 10.0 a.m.)^36^. Our results imply that *R. longifrons* life stages can be released at any time of the day and we recommend the release of the predator third-stadium for *A. gossypii* control in screen house.

The predominance of *A. gossypii* and *P. solenopsis* populations in cotton fields respectively during south-west and post-monsoon seasons are not surprising. In fact, it is known that the population dynamics of aphids and mealybugs can be affected by seasonal changes in weather conditions^37^. Moreover, Wang *et al*.^38^ showed that high rainfall was unfavourable for serious infestation by *P. solenopsis*. Our study showed that, augmentative releases of *R. longifrons* significantly reduced the number of insect pests in the cotton fields. The first release of this predator in cotton fields was capable to reduce *A. gossypii* and *P. solenopsis* populations during south-west and post monsoon compared with the control. Similarly, the field release of some reduviid predators such as *Pristhesancus plagipennis* Walker^17,39^, *Platymeris laevicollis* Distant^40^ and *Rhynocoris marginatus* (Fab.)^19,20^ were successful in reducing various pests in coconut palm, cotton and groundnut fields. Our results showed that *R. longifrons* can be an effective predator of multiple preys, which is consistent with previous results for generalist predators^9,41,42^.

Further, no adverse interaction between *R. longifrons* and indigenous predators like coccinellids, spiders and, other reduviids were observed. This situation suggests reduviid predators do not interfere with other natural enemies. Consequently, reduviid predator may be an ecofriendly protection for cotton pest populations and are likely to be highly detrimental to their biological control. Reduviids can be utilized as part of a multiple species release program as suggested for *Dephastus catalinae* (Horn) (Coleoptera: Coccinellidae)^43^. However, Symondson *et al*.^44^ reported that at natural ecosystems, change of biotic and abiotic variables is common and that under such circumstance, it was difficult to predict the interspecific and intraspecific interactions between released generalist predators, and the predators dwelled in the eco-system. The decrease of ants in *R. longifrons* treated plots could be explained among other factors, by the decrease of aphids and mealybugs populations, which bribe them with their honeydew excretion. In fact, it is known that *P. solenopsis*^45^ and, *A. gossypii*^46^ present a mutualism association with ants.

Comparatively of the cotton production (1585 kg/ha) at Tamil Nadu of India, the yield recorded during our study in cotton fields during south-west monsoon (2068 kg/ha) and post-monsoon season (1862 kg/ha) reveal that release of reduviids reduced impact of insect pests thus enhance cotton production. Similar trends were also observed when *P. plagipennis* was release in cotton fields^17,39^. Although the cost benefit ratio was low (1:1.28 or 1:1.24) comparatively to entomopathogenic insecticides such as HaNPV (1:3.50) or Bt (1:1.23) used for cotton protection^47,48^, it had been higher than those of control highlighting the benefit of integrating this predator in cotton pest management. Furthermore, the small-scale mass production technology for *R. longifrons* developed by Sahayaraj and Ravi^49^ is available for an augmentative biological control program. In view of these encouraging results, it would be interesting in the framework of a biocontrol of cotton pests program to integrate this predator in other agro-climatic zones in Tamil Nadu as well as in other states in India.

## Conclusion

Based on the observed hiding behaviour of *R. longifrons*, we conclude that this predator can survive and adapt to the cotton agro-ecosystem after its release. In the framework of an IPM program, *R. longifrons* life stages can be releases at any time of the day. Augmentative releases of this predator reduced significantly the number of insect pests in the cotton fields. *R. longifrons* increased the cotton production and enhanced the cost benefit ratio increased in predator released cotton field. It is concluded that reduviid predators can be integrated into cotton integrated pest management program.

## Methods

### Collection and maintenance of insects

Life stages of *R. longifrons* were collected from cotton fields (2 females, 1 male, 5 nymphs) and scrub jungle (3 females, 2 males, and 8 nymphs) bordering agro-ecosystems (8.7038° N, 77 0.8625° E) in Tirunelveli districts of Tamil Nadu, India. They were maintained on a factitious host, fourth and fifth instar *Corcyra cephalonica* (Stainton) (Lepidoptera: Pyralidae) under laboratory conditions at 30 ± 2 ^o^C, 61 ± 5% relative humidity (RH) and photoperiod of 11 L:13 D hr in 1 L transparent plastic containers (15 cm diameter × 8 cm height) as described by Sahayaraj *et al*. (2002). *Corcyra cephalonica* eggs were purchased from the Agriculture Office, Palayamkottai, and maintained in 2 L plastic troughs (20 cm diameter × 10 cm height) on crushed wheat and groundnuts. For conducting various studies, *R. longifrons* life stages, third (26.8 ± 0.02 mg), fourth (35.4 ± 0.03 mg), fifth (54.8 ± 0.07 mg) nymphal instars, and adults (male = 67.8 ± 0.08 mg, female = 76.8 ± 0.09 mg) were used.

Adults and immatures of the *A. gossypii*, *P. solenopsis, D. cingulatus*, and eggs and larvae of *H. armigera* were collected from cotton and maintained on their natural host plant under the above mentioned laboratory conditions. Life stages of *D. cingulatus* were maintained on wet cotton seeds. Both *A. gossypii* and *P. solenopsis* life stages were maintained on cotton seedlings (5–10 days old) which were established in small plastic tubs (10 cm height × 8 cm upper diameter × 6 cm lower diameter). On each seedling 40–50 *D. cingulatus* nymphs or adults were accommodated. *H. armigera* larvae were maintained individually in plastic containers (8 × 6.5 cm) with a mixture of healthy cotton flowers, flower buds and young leaves.

### Hiding behaviour of the predator inside the screen houses

To record the hiding places of the predator when it was released in cotton during an augmentative pest management programme, the methodology described by Tomson *et al*.^31^ was used. Observations of hiding behaviour of *R. longifrons* were carried out in a screen house (12 m length × 7.8 m width) using 30 day-old cotton plants (variety: MCU-5). The plot consisted of 52 plants (4 column × 13 rows) with inter-plant spacing of 0.60 m and inter-row distances of 0.75 m, oriented east to west. Before releasing the different life stages of the *R. longifrons*, the cotton plants, fallen leaves, pebbles, and other objects found in the plots were checked thoroughly to confirm the absence of any reduviids. Then, 10 fourth instar predators (previously starved for 24 hr) were released at 6.00 a.m., 8.30 a.m., 11.00 a.m. and 3.30 p.m in different pots. The reduviids were introduced individually by means of a camel’s hair brush into the northeast corner of the base of the plant. Two hours after every release, the percentage of insects that settled at various places [cotton plant, fallen leaves, small pebbles in the plant and other objects (soil balls, weed plants)] were recorded. Similar procedure was followed for the fifth instars and adult predators.

### Biocontrol potential of *R. longifrons* in relation to day and night hours

Modified methodology of Tomson *et al*.^31^ was followed for bioefficacy experiments. For the experiments, cotton seeds of MCU-5 variety were sown in cement pots (36 × 30 × 22 cm) and maintained in screen house at St. Xavier’s College, Palayamkottai. The bioefficacy of *R. longifrons* life stages (third, fourth and fifth instar nymphs and adults) was tested against preferred life stages of *A. gossypii* (4 adults/predator)*, D. cingulatus* (second and third nymphal instars, 2 of each instar/predator), *H. armigera* (second and third instar larva, 2 of each instar/predator) and *P. solenopsis* (4 adults/predator). The predatory potential of *R. longifrons* was evaluated between 6 a.m. and 6 p.m. (day experiments) and between 6 p.m. and 6 a.m. (night experiments). pests were released on the 25–30-day old cotton plants covered by a nylon net at 6 a.m. for day or 6 p.m for night experiments respectively. One hour after pests release, 24-hour pre-starved *R. longifrons* (third, fourth and fifth instar nymphs, and adults - 2/plant) were introduced separately on the infested cotton plants. Six replications were used for each pest and predator life stage for both experiments. Three replications without predator were established as control. After 12 h, the number of live and dead preys in each plant was counted and predation rate of the *R. longifrons* life stages was calculated^50^.

### Experimental design for the augmentative biocontrol potential evaluations

The augmentative biocontrol potential was evaluated during rain fed condition (South-west monsoon) 2011 (July to September) in the cotton field at Kothankulam (9.4692° N, 77.6046° E), in the Virudhunagar district, Tamil Nadu and also in irrigated condition (post-monsoon season) 2011–2012 (December to February) at K. Duraisamiyapuram (E 77° 35°, N 22.16°), in the Tuticorin district of Tamil Nadu. The methodology described by Tomson *et al*.^31^ with slight modifications was used for these evaluations. For weed control and fertilization farmers’ standard cultural practices were used to grow cotton (SVPR4 cultivar). Two treatments were evaluated, a cotton field into which *R. longifrons* was released and a control field, free of pesticides and predators. Unplanted buffer zone (2 m) was established between treatment and control plots. The treatment plots were arranged in randomized complete block design with five replications (10 plots total with a size of 10 m × 5 m each). To identify the predominant pests and predators in experimental cotton fields, we examined in each field 8–10 leaves of 10-randomly selected plants from two days before predators release. *R. longifrons* nymphal instars (50 individuals each) were released individually during 6–8 a.m. in each experimental cotton fields 40, 55 and 70 days after the seedling emergence (ASE). In addition, three cards (3 × 3 cm) containing each 50 eggs of *R. longifrons* were also placed onto the twigs of a plant in each cotton field. In total, a mixture of 900 *R. longifrons* (150 eggs, 250 nymphal instars × 3 releases = 900/plot) were released in each plot. Three days after the predators’ release, 10 randomly selected cotton plants in each experimental cotton field were visually examined for the presence of *A. gossypii, D. cingulatus, P. solenopsis* and *H. armigera*. The number of each species of predators encountered was also recorded per plant.

### Cost Benefit Ratio (CBR) and Percent Avoidable loss

At the completion of the growing seasons, the cotton in each plot was harvested. It was then cleaned, weighed and sold in the local market. The cost benefit ratio (CBR) was calculated based on the income per hectare thus generated. In addition, the percent of avoidable loss (PAL) was also calculated according to the method proposed by Krishnaiah^51^.

### Statistical analysis

Before variance analysis, the data normality was tested using Levene’s test. Data on the percentage of predator hiding under various objects in relation to life stages was subjected to arcsine transformation, while the total number of prey consumed by a predator in 24 h, numbers of different insect pests and natural enemies present at each release (first, second and third), as well as their total mean populations were log-transformed to homogenize the variances before being subjected to variance analysis using IBM SPSS Statistics version 25 software package. Significant differences between the means were separated using the Student Newman Keuls test (p ≤ 0.05). Original means are presented in tables and figures.
